# P-166. Safety and Efficacy of Colchicine in Covid-19 Treatment: Systematic Review and Meta-Analysis

**DOI:** 10.1093/ofid/ofaf695.390

**Published:** 2026-01-11

**Authors:** Ahmed Hosney Nada, Ismail A Ibrahim, Nada Khalid Asar, Abdulrahman Qenawy, Mariam Mahmoud Mohammed, Mohamed Wagdy, Heidi Sherif Farouk

**Affiliations:** Faculty of Medicine, Benha University, Egypt, Quesna, Al Minufiyah, Egypt; Fenerbahce University, Faculty of Health Sciences, Istanbul, Istanbul, Turkey; Mansoura university, Giza, Al Jizah, Egypt; Faculty of Medicine, South Valley University, Qena, Egypt, Qina, Qina, Egypt; Faculty of Pharmacy, Fayoum University, Fayoum, Egypt, Al fayyum, Al Fayyum, Egypt; modern university for technology and information,Cairo,Egypt, basil, Kafr ash Shaykh, Egypt; Faculty Of medicine, Alexanderia University, Egypt, Alexanderia, Al Iskandariyah, Egypt

## Abstract

**Background:**

The COVID-19 pandemic has prompted exploration of various therapeutic agents, including colchicine, due to its anti-inflammatory properties. This systematic review and meta-analysis evaluate the efficacy and safety of colchicine in managing COVID-19.

**Methods:**

A comprehensive literature search was conducted across PubMed, Cochrane, Scopus, Web of Science, and Embase till March 2024.Primary outcomes were 28-day mortality, invasive and non-invasive mechanical ventilation, and ICU admission. Secondary outcomes were inflammatory markers (CRP, D-dimer, IL-6, LDH, Ferritin), ICU length of stay, and hospitalization duration.

**Results:**

17 randomized controlled trials with a total of 25478 patients were included. Colchicine did not significantly reduce 28-day mortality (RR = 1.00, 95% CI [0.93 : 1.07], P = 0.94), invasive mechanical ventilation (RR = 1.03, 95% CI [0.93 : 1.15], P = 0.58), non-invasive ventilation (RR = 0.81, 95% CI [0.44 : 1.48], P = 0.49), or ICU admission (RR = 0.89, 95% CI [0.56 : 1.41], P = 0.62). There was no significant reduction in ICU stay or hospitalization duration. While colchicine showed a reduction in CRP and D-dimer levels after sensitivity and subgroup analysis, no consistent anti-inflammatory benefits were observed across all biomarkers.

**Conclusion:**

Colchicine does not provide significant clinical benefits in COVID-19 management. Further large-scale RCTs with standardized dosages and long-term follow-up are required to clarify its role.

**Disclosures:**

All Authors: No reported disclosures

